# Long non‐coding RNA RACGAP1P promotes breast cancer invasion and metastasis via miR‐345‐5p/RACGAP1‐mediated mitochondrial fission

**DOI:** 10.1002/1878-0261.12866

**Published:** 2020-12-16

**Authors:** Danmei Zhou, Kehan Ren, Meili Wang, Jigang Wang, Ermin Li, Chenjian Hou, Ying Su, Yiting Jin, Qiang Zou, Ping Zhou, Xiuping Liu

**Affiliations:** ^1^ Department of Pathology School of Basic Medical Sciences Fudan University Shanghai China; ^2^ Department of Pathology Shanghai Fifth People's Hospital Fudan University Shanghai China; ^3^ Department of Pathology The Affiliated Hospital of Qingdao University China; ^4^ Department of Physiology and Pathophysiology School of Basic Medical Sciences Fudan University Shanghai China; ^5^ Department of General Surgery Huashan Hospital Fudan University Shanghai China

**Keywords:** breast cancer, metastasis, MiR‐345‐5p, mitochondrial fission, RACGAP1, RACGAP1P

## Abstract

Long non‐coding RNAs (lncRNAs) are emerging as key molecules in various cancers, yet their potential roles in the pathogenesis of breast cancer are not fully understood. Herein, using microarray analysis, we revealed that the lncRNA RACGAP1P, the pseudogene of Rac GTPase activating protein 1 (RACGAP1), was up‐regulated in breast cancer tissues. Its high expression was confirmed in 25 pairs of breast cancer tissues and 8 breast cell lines by qRT‐PCR. Subsequently, we found that RACGAP1P expression was positively correlated with lymph node metastasis, distant metastasis, TNM stage, and shorter survival time in 102 breast cancer patients. Then, *in vitro* and *in vivo* experiments were designed to investigate the biological function and regulatory mechanism of RACGAP1P in breast cancer cell lines. Overexpression of RACGAP1P in MDA‐MB‐231 and MCF7 breast cell lines increased their invasive ability and enhanced their mitochondrial fission. Conversely, inhibition of mitochondrial fission by Mdivi‐1 could reduce the invasive ability of RACGAP1P‐overexpressing cell lines. Furthermore, the promotion of mitochondrial fission by RACGAP1P depended on its competitive binding with miR‐345‐5p against its parental gene RACGAP1, leading to the activation of dynamin‐related protein 1 (Drp1). In conclusion, lncRNA RACGAP1P promotes breast cancer invasion and metastasis via miR‐345‐5p/RACGAP1 pathway‐mediated mitochondrial fission.

## Graphical abstract

In this study, we illustrate the potential mechanism of RACGAP1P implicated in breast cancer invasion and metastasis. In breast cancer cells, RACGAP1P could competitively bind to miR‐345‐5p, which targets RACGAP1, and therefore up‐regulate RACGAP1. RACGAP1P overexpression promoted mitochondrial fission‐mediated cell invasion via the RACGAP1P/miR‐345‐5p/RACGAP1/Drp1 network.

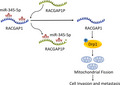

**Abbreviations**
CDS: coding sequence
ceRNAs: competitive endogenous RNAs
Drp1: dynamin‐related protein 1
FFPE: formalin‐fixed paraffin‐embedded
lncRNAs: long non‐coding RNAs
miRNAs: microRNAs
RACGAP1: Rac GTPase activating protein 1
TCGA: The Cancer Genome Atlas

## 1 Introduction

There are about 279 100 new cases diagnosed with breast cancer and 42 690 estimated deaths due to breast cancer in the United States in 2020. Breast cancer is still the second leading cause of cancer‐related death in women [1]. Most of the patients of the terminal stage present with tumors metastasized to the lung, liver, brain, or bone, compromising organ function and leading to patient death [2]. Metastasis is a complex, multistep biological process, involving a multitude of genes and biomolecules, and determinant for the death of breast cancer patients [3]. Many studies have investigated the molecular mechanism underlying this malignant biological process, but the knowledge of its mechanisms is still fragmentary and needs to be broadened in order to improve the therapeutic approach.

Recent studies suggested that mitochondrial fission was involved in cancer cell invasion and metastasis, and the inhibition of mitochondrial fission could suppress tumor metastasis [4, 5, 6]. Mitochondria exist as dynamic networks maintained by two processes: fission and fusion, regulated by dynamin‐related protein 1 (Drp1) and mitofusins (Mfns), respectively [7, 8]. Altered levels of phosphorylation or expression of Drp1 are tightly linked to the unbalance of mitochondrial fission and fusion [9]. However, its upstream regulatory mechanism is still unknown, which is crucial to elucidate the role of mitochondrial fission in the cancer metastasis.

Long non‐coding RNAs (lncRNAs), a subgroup of non‐coding RNAs, are non‐protein‐coding transcripts with more than 200 nucleotides in length [10]. lncRNAs are transcribed abundantly in mammalian cells and interact with many known cancer genes [11]. Accumulating evidence has shown that lncRNAs are important players in the genesis and progression of human cancers, such as breast cancer, colon cancer, prostate cancer, and liver cancer, which may serve as effective diagnostic and therapeutic targets [12, 13, 14, 15]. Although thousands of lncRNAs have been found, only a few of them have been functionally characterized. As a specific subgroup of lncRNA, pseudogenes, can interact with their corresponding parental genes by competing for the same microRNAs (miRNAs), function as competitive endogenous RNAs (ceRNA) [16, 17]. LncRNA RACGAP1P is the pseudogene of Rac GTPase activating protein 1(RACGAP1), which is a member of GTPase activating protein family. RACGAP1P has been reported to elicit its oncogenic activity as a ceRNA to competitively sequestrate miR‐15‐5p from its endogenous target RACGAP1, thereby leading to the activation of RhoA/ERK signaling and contributing to the recurrence of hepatocellular carcinoma [18]. However, whether RACGAP1P, mitochondrial fission, and breast cancer metastasis have certain crosstalk remains unknown; and the explanation of the related mechanism can provide novel therapeutic strategies for breast cancer.

In this study, we reported the identification of lncRNA RACGAP1P (NR_026583) in breast cancer. We found that RACGAP1P was overexpressed in breast cancer tissues, and closely related to tumor metastasis and poor prognosis of breast cancer patients. Then, we further clarified the regulatory mechanism of RACGAP1P in breast cancer metastasis induced by mitochondrial fission in a miR‐345‐5p/RACGAP1‐dependent manner.

## 2 Materials and methods

### 2.1 Clinical specimens

All patients diagnosed as breast cancer were recruited in Huashan Hospital Affiliated to Fudan University. Three independent cohorts of patients were enrolled in the study. Cohort 1 included five breast cancer and corresponding matched normal tissues, which were used for microarray and detailed in Table S1. Cohort 2 included 25 breast cancer samples and paired adjacent non‐cancerous tissues from January 2011 to July 2012, and Cohort 3 included 102 formalin‐fixed paraffin‐embedded (FFPE) breast cancer tissues from January 2008 to September 2008. The samples in Cohort 1 and Cohort 2 dissected surgically were immediately stored in liquid nitrogen until use. None of the patients had been pretreated with chemotherapy or radiotherapy before mastectomy. Written Informed consent was obtained from all patients. The study protocol followed the standards of the Declaration of Helsinki, and the study was approved by the Ethics Committee of Shanghai Medical College, Fudan University.

### 2.2 Animals

Female nude mice (aged 4–6 weeks, *n* = 20) were obtained from Shanghai SLAC Laboratory Animal Co. Ltd. (Shanghai, China). All experimental procedures followed the guidelines of the National Institutes of Health Guide for The Care and Use of Laboratory Animals and were approved by the Ethics Committee of Shanghai Medical College, Fudan University. 2 × 10^6^ cells were injected into the tail vein of each nude mice. Eight weeks after injection, mice were euthanized, and organs were immediately removed and processed for histological evaluation.

### 2.3 Cell lines

The cell lines MCF10A, T47D, MCF7, MDA‐MB‐468, MDA‐MB‐453, BT474, BT549, and MDA‐MB‐231 were obtained from the Type Culture Collection of Chinese Academy of Sciences (Shanghai, China). The specific culture conditions of cells referred to ATCC (https://www.atcc.org/) and the specifications of breast cancer cell lines were summarized in Table S2 [19, 20].

### 2.4 Microarray analysis

Briefly, samples (five breast cancer tissues and matched normal tissues) were used to synthesize cDNA with Quick Amp Labeling kit (Agilent, Palo Alto, CA, USA). cDNA was labeled and hybridized to the Human LncRNA Microarray v2.0 (Arraystar, Rockville, MD, USA). After hybridization, processed slides were scanned using the Agilent DNA Microarray Scanner (Agilent p/n G2565BA). Agilent Feature Extraction Software v. 11.5.1.1 was applied to analyze the acquired array images. Agilent GeneSpring GX v11.5.1 was used to normalize the quantile and subsequently process the data. Differentially expressed lncRNAs were identified through Volcano Plot filtering. The threshold of up‐ and down‐regulated lncRNAs was a *P* value < 0.05 and a fold change > 1.5.

### 2.5 Cell proliferation assay

Cell counting kit‐8 (CCK‐8; Beyotime Biotechnology, Shanghai, China) was adopted to detect the cell proliferative ability. Briefly, MDA‐MB‐231 and MCF7 cells (5 × 10^3^ cells/well) were seeded into 96‐well plates. At indicated time points, 10 μL CCK‐8 solution was added to each well and then continued to incubate for 2 h at 37 °C. Finally, the spectrophotometric absorbance was detected at 450 nm.

### 2.6 Wound‐healing assay

MDA‐MB‐231 and MCF7 cells were seeded into 6‐well plates. Until 100% confluency, the scratch was created using a plastic pipette tip. Then, the cells were washed with PBS three times and incubated with serum‐free medium. For MDA‐MB‐231, the images were taken at 0, 24, and 36 h; for MCF7, the images were taken at 0, 36, and 72 h, using a microscope, respectively.

### 2.7 Transwell assay

For invasion analysis, 24‐well transwell chambers (Corning, Corning, NY, USA) were pre‐coated with Matrigel. Subsequently, 1 × 10^4^ cells in 200 μL serum‐free medium were seeded per well in the upper chambers, and 500 μL medium with 10% FBS was placed to the lower chambers. After culturing at 37 °C for 24 h, the invaded cells were fixed and stained. Five random fields were selected to count using imagej software (National Institutes of Health, Bethesda, MD, USA). For migration analysis, transwell chambers were employed without the Matrigel matrix.

### 2.8 Quantitative real‐time PCR (qRT‐PCR)

Total RNA from fresh tissues and cells was extracted using TRIzol Reagent (Invitrogen, Carlsbad, CA, USA), following the manufacturer's instructions. Total RNA from FFPE tissue sections was extracted using the RecoverAll™ Total Nucleic Acid Isolation Kit (Invitrogen). After cDNA synthesization by Prime Script RT Master Mix (TaKaRa, Dalian, China), real‐time PCR was carried out using SYBR Green PCR Master Mix (Invitrogen). The primers are listed in Table S3. Notably, for paraffin‐embedded tissues, we designed three primers to amplify short amplicons (~ 60 bp) as described previously [21]. GAPDH was used as an internal control for mRNA and lncRNA, and miRNA expression was normalized to U6 expression.

The expression level of objective gene was obtained using the calculation as 2^‐ΔΔCt^ [ΔCt = Ct (objective gene) − Ct (internal control gene), ΔΔCt = ΔCt (experimental group) − ΔCt (control group)], where Ct value represented the threshold cycle for each transcript. For 102 FFPE samples, the expression levels of RACGAP1P were obtained using the calculation as 2^−ΔCt^ [ΔCt = Ct (RACGAP1P) − Ct (GAPDH), and the Ct value was the mean value of the results for three different primers. Moreover, the median of RACGAP1P expression was considered to be the cutoff value of 102 breast cancer.

### 2.9 Western blot

Whole‐cell lysates were prepared using RIPA buffer, and equivalent amounts of protein (from 10 to 30 μg) were resolved by SDS/PAGE. Proteins were transferred to poly(vinylidene difluoride) membranes (Millipore, Billerica, MA, USA) and immunoblotted with the indicated antibodies as follows: RACGAP1 (1 : 1000; AbSci, #AB32752, Nanjing, China), Drp1 (1 : 1000; CST, #5391, Danvers, MA, USA), p‐Drp1 (Ser161) (1 : 1000; CST, #4494), GAPDH (1 : 1000; Santa Cruz, # sc‐51097, Santa Cruz, CA, USA), Mfn1 (1 : 1000; Santa Cruz, # sc‐166644, ), Mfn2 (1 : 1000; CST, #9482). After overnight at 4 °C, the membranes were washed thrice and then incubated with the secondary horseradish peroxidase‐linked antibodies as follows: anti‐mouse antibody (1 : 1000; CST, #7076), anti‐rabbit antibody (1 : 1000; CST, #7074).

### 2.10 Plasmids and lentiviral transduction

Full‐length RACGAP1P cDNA, RACGAP1 gene coding sequence (CDS) cDNA, and shRNA‐resistant RACGAP1 cDNA (Table S4) were synthesized and cloned into pCDH‐CMV‐MCS‐EF1‐Puro, respectively. The pLKO.1‐puro was modified by replacing the puromycin‐resistant gene with a neomycin‐resistant gene to generate pLKO.1‐neo plasmid. The figures of the lentivirus vector with all the sites specification are displayed in Fig. S1. RACGAP1 shRNA sequence (Table S5) was cloned into modified pLKO.1‐neo. HEK‐293T cells were seeded to produce lentivirus particles using lipofectamine 2000 (Invitrogen). Forty‐eight hours after transfection, the conditioned medium was centrifuged, and the supernatant was then applied to target cells supplemented with puromycin or G418 selection.

### 2.11 Dual‐luciferase reporter assay

MDA‐MB‐231 and MCF7 cells were transfected with miR‐345‐5p mimic or miR‐345‐5p control, respectively, and with RACGAP1P/RACGAP1 luciferase reporter and Renilla control luciferase vector (Promega, Madison, WI, USA). The luciferase activities were detected by the Dual‐luciferase reporter assay system (Promega), according to the manufacturer's protocol.

### 2.12 RNA immunoprecipitation (RIP)

The Magna RIP™ RNA‐Binding Protein Immunoprecipitation Kit (Millipore) was used to conduct RIP assays following the manufacturer's instructions. Briefly, MDA‐MB‐231 and MCF7 cells at 90% confluency were harvested using RIP lysis buffer. The cell lysates were incubated with RIP buffer containing magnetic beads conjugated with anti‐ Ago2 antibody (Abcam, Cambridge, MA, USA) or negative control IgG (Millipore). Then, proteinase K was added to digest the protein, and the supernatants containing RNA were extracted and subjected to qRT‐PCR.

### 2.13 Quantification of mitochondrial interconnectivity

Cells were stained by MitoTracker Red (Thermo Fisher Scientific; 100 nm, 45 min; Waltham, MA, USA) at 37 °C. After being washed in PBS, cells were fixed in 3.7% formaldehyde and then sealed with 75% glycerin for microscopy. Cell nuclei were visualized with DAPI. Images were acquired by a Carl Zeiss (Jena, Germany) AX10 microscope and then analyzed using imagej (National Institutes of Health) with Mito‐Morphology Marco Plug‐in [22]. Mitochondrial interconnectivity was determined by pixel area/perimeter.

### 2.14 Statistics

All statistical analyses were performed using spss 21.0 software (IBM Corp., Armonk, NY, USA). Comparisons between two groups were analyzed by the Student's *t*‐test. Data among three groups were analyzed by one‐way analysis of variance (ANOVA). The chi‐square test was used for the analysis of clinicopathologic relevance of RACGAP1P. Pearson *r* method was applied to gene correlation expression analysis. The diagrams were completed with prism 6 (GraphPad Software, San Diego, CA, USA). A value of *P* < 0.05 was considered to be statistically significant.

## 3 Results

### 3.1 RACGAP1P is up‐regulated in breast cancer and associated with poor prognosis

**Fig. 1:**
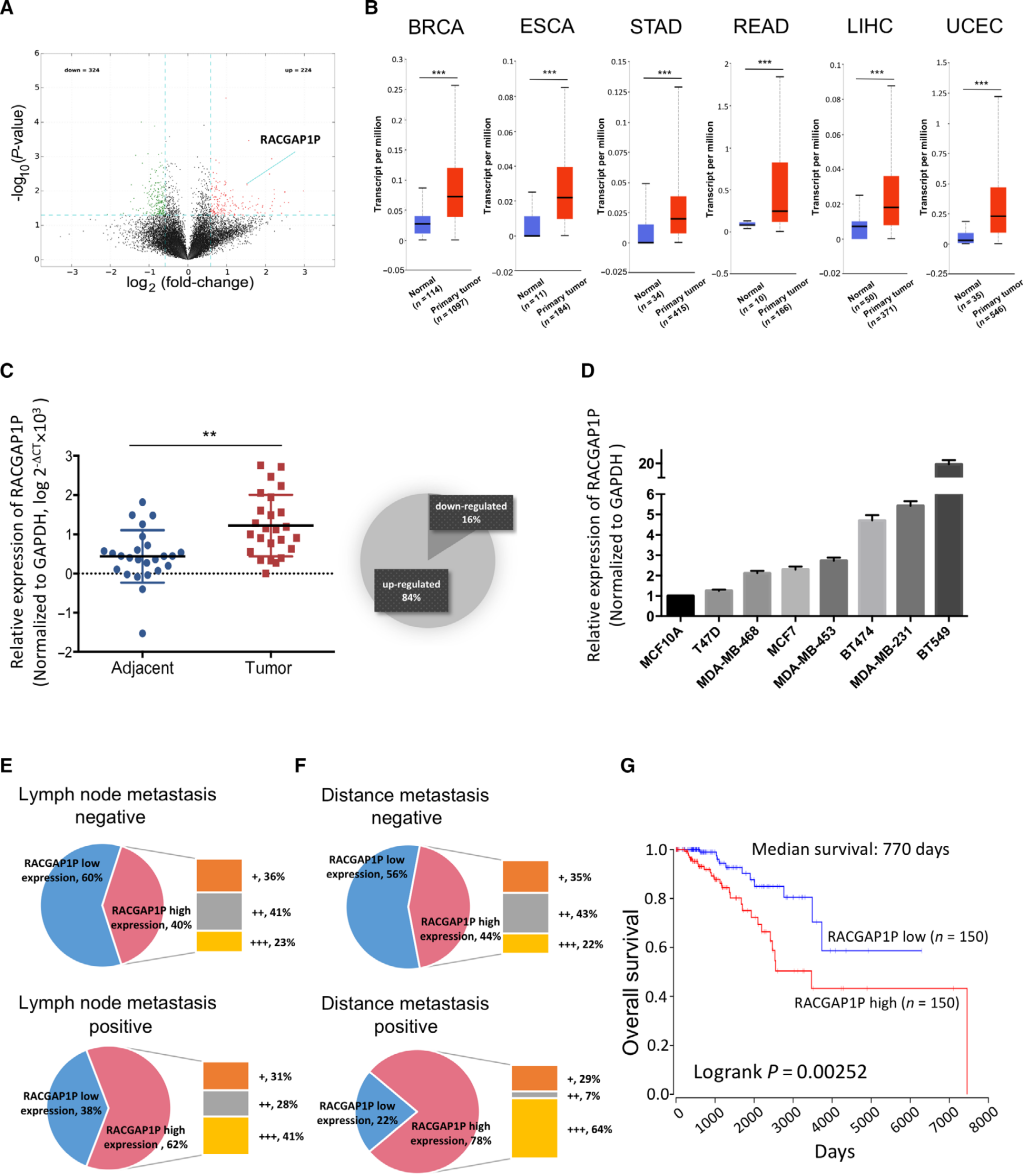
Overexpression of RACGAP1P in Breast Cancer. (A) The volcano plot lists differently expressed lncRNAs (fold change >1.5; *P* < 0.05) between five breast cancer tissues and matched adjacent non‐cancerous tissues. (B) The mRNA expression level of RACGAP1P was up‐regulated in six types of human cancer, performed on UALCAN online software. BRCA, breast invasive carcinoma; ESCA, esophageal carcinoma; STAD, stomach adenocarcinoma; READ, rectum adenocarcinoma; LIHC, liver hepatocellular carcinoma; UCEC, uterine corpus endometrial carcinoma. (C) RACGAP1P expression level was analyzed by qRT‐PCR in 25 breast cancer tissues compared to matched non‐cancerous breast tissues. The expression intensity was normalized as log 2^−ΔCT^ × 10^3^. **P < 0.01. (D) RACGAP1P expression level was analyzed by qRT‐PCR in normal breast epithelial cell line MCF10A and seven kinds of breast cancer cell lines. MCF10A vs. T47D: *P* = 0.9872, 95% CI: −1.529 to 1.018; MCF10A vs. MDA‐MB‐468: *P* = 0.0988, 95% CI: −2.390 to 0.1573; MCF10A vs. MCF7: *P* = 0.0450, 95% CI: −2.571 to −0.02350; MCF10A vs. MDA‐MB‐453: *P* = 0.0061, 95% CI: −3.006 to −0.4585; MCF10A vs. BT474: *P* < 0.0001, 95% CI: −4.977 to −2.430; MCF10A vs. MDA‐MB‐231: *P* < 0.0001, 95% CI: −5.704 to −3.157; MCF10A vs. BT549: *P* < 0.0001, 95% CI: −19.95 to −17.41. One‐way ANOVA. (E) RACGAP1P mRNA expression in lymph node metastasis negative and lymph node metastasis positive breast cancer patients was shown in the pie chart. Further, the absolutely relative mRNA expression of RACGAP1P (calculation as 2^−ΔCt^) in RACGAP1P high expression groups was arranged from small to large. Then, RACGAP1P expression was defined as + (bottom 33%), ++ (middle 33%), +++ (top 33%). (F) RACGAP1P mRNA expression in distance metastasis negative and distance metastasis positive breast cancer patients was shown in the pie chart, using the same method with (E). (G) Kaplan–Meier curve in breast cancer patients was generated using the OncoLnc resource. TCGA data of 1006 breast cancer patients were assigned into two groups by percentile of RACGAP1P expression level (top 15% as high expression group, bottom 15% as low expression group). Log‐rank test, *P* = 0.00252.

To better reveal the roles of lncRNAs in breast cancer, we extracted RNA from five pairs of breast cancer tissues and matched adjacent non‐cancerous tissues and then did the transcriptome microarray analysis. From 22 213 denoted lncRNAs, 548 lncRNAs with fold change > 1.5 and *P* < 0.05 were identified differentially expressed in breast cancer tissues compared to adjacent tissues (Fig. 1A). Among 224 up‐regulated lncRNAs, we focused on a pseudogene, RACGAP1P, which was confirmed overexpressed in various human cancers compared with normal tissues using data from The Cancer Genome Atlas (TCGA) (http://ualcan.path.uab.edu) (Fig. 1B). qRT‐PCR analysis was applied to detect the expression of RACGAP1P in 25 pairs of breast tissues (cancer and adjacent non‐cancerous tissues) and 8 breast cell lines (normal breast epithelial cell line and breast cancer cell lines). The results showed RACGAP1P was consistently up‐regulated in both breast cancer tissues (Fig. 1C) and breast cancer cells (Fig. 1D). Furthermore, PhyloCSF analysis [23] demonstrated that RACGAP1P was unlikely to present a protein‐coding region (Fig. S2A).

To elucidate the clinicopathologic significance of RACGAP1P in breast cancer, we subsequently detected the expression levels of RACGAP1P in 102 breast cancer FFPE samples by qRT‐PCR. The subtypes were defined according to the St. Gallen Consensus 2013 [24]. As shown in Table 1, RACGAP1P expression was positively correlated with lymph node metastasis (*P* = 0.0183), distance metastasis (*P* = 0.0224), and TNM stage (*P* = 0.0036). Further analysis depicted higher RACGAP1P expression accounted for a larger proportion of patients with lymph node metastasis (Fig. 1E) and distance metastasis (Fig. 1F). Moreover, OncoLnc online Kaplan–Meier analysis (http://www.oncolnc.org) using TCGA data (Network, 2012) revealed that breast cancer patients with a high expression level of RACGAP1P had significantly shorter survival time (Fig. 1G).

**Table 1:** Relationship between RACGAP1P and clinicopathological factor (*n* = 102).

Characteristics	Number	RACGAP1P expression		*P* values
		Low	High	
Age (years)				
< 50	59	31	28	0.5475
≥ 50	43	20	23	
Tumor size (cm)				
≤ 2	75	39	36	0.5008
> 2	27	12	15	
Lymph node metastasis				
Negative (−)	55	33	22	**0.0289**
Positive (+)	47	18	29	
Distance metastasis				
Negative (−)	84	47	37	**0.0094**
Positive (+)	18	4	14	
TNM classification				
Ⅰ	43	26	17	**0.0239**
Ⅱ	41	21	20	
Ⅲ	18	4	14	
Histological grade				
Ⅰ (well)	30	14	16	0.4650
Ⅱ (moderate)	46	26	20	
Ⅲ (poor)	26	11	15	
ER status				
Negative (−)	39	20	19	0.8385
Positive (+)	63	31	32	
PR status				
Negative (−)	46	21	25	0.4261
Positive (+)	56	30	26	
HER2 status				
Negative (−)	66	35	31	0.4072
Positive (+)	36	16	20	
Ki67 status				
Negative (−)	28	15	13	0.6572
Positive (+)	74	36	38	
Subtype				
Luminal A	19	12	7	0.5346
Luminal B	44	19	25	
HER2‐positive	19	10	9	
Triple negative	20	10	10	

Bold values indicated *P*‐value with statistical significance.

### 3.2 Overexpression of RACGAP1P promotes breast cancer metastasis

**Fig. 2:**
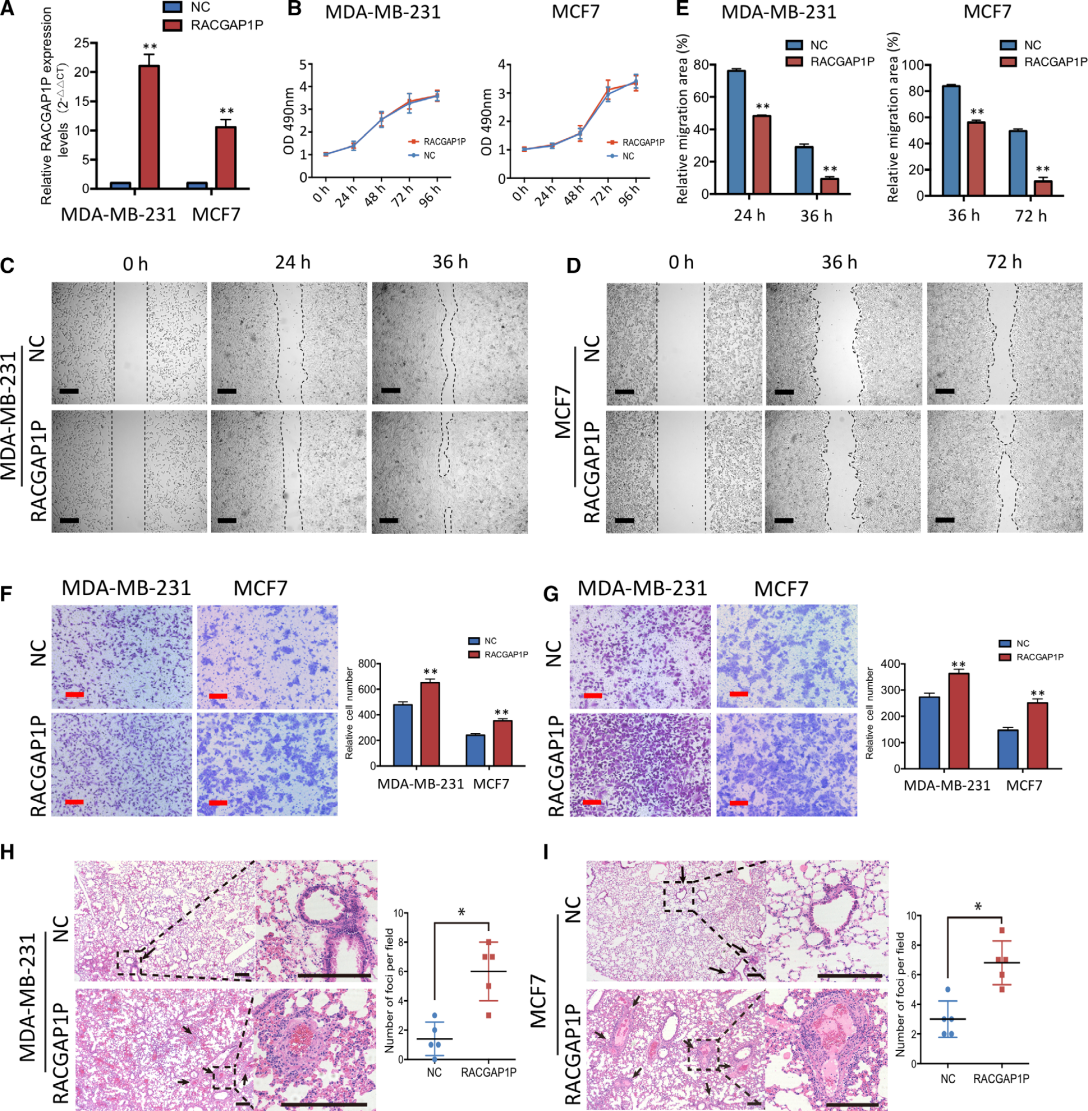
RACGAP1P promoted breast cancer cell migration and invasion. (A) RACGAP1P expression was detected in MDA‐MB‐231 and MCF7 cells by qRT‐PCR after transfection of lentivirus harboring the full‐length human RACGAP1P sequence or the empty vector. **P < 0.01. (B) The proliferation of MDA‐MB‐231 and MCF7 cells was detected by CCK8 assays. (C and D) The cell migration capability of MDA‐MB‐231 and MCF7 cells was assessed by wound‐healing assays at specific time points as indicated. Scale bar (black), 20 μm. (E) Scratch wound area of MDA‐MB‐231 and MCF7 cells was measured by imagej software, and relative migration area was presented by spss software. **P < 0.01. (F) The cell migration capability of MDA‐MB‐231 and MCF7 cells was assessed by transwell migration assays at 48 h. Scale bar (red), 50 μm. **P < 0.01. (G) The cell invasion ability of MDA‐MB‐231 and MCF7 cells was analyzed by transwell invasion assays at 48 h. Scale bar (red), 50 μm. **P < 0.01. (H and I) Hematoxylin and eosin‐stained images (left) of lung tissue isolated from nude mice that received intravenous tail injections of scramble or RACGAP1P overexpressed cells. Arrows indicated the metastasis foci. The number of tumor nodules per high power field (right) was quantified from the stained lung sections; *n* = 5. *P < 0.05. Error bars, SD; Student’s *t*‐test.

To further evaluate the function of RACGAP1P in breast cancer, we stably overexpressed RACGAP1P in MDA‐MB‐231 breast cancer cells (MDA‐MB‐231 RACGAP1P) and MCF7 breast cancer cells (MCF7 RACGAP1P), verified by qRT‐PCR (Fig. 2A). We found RACGAP1P overexpression did not affect breast cell proliferation (Fig. 2B), followed by no significant difference in tumor cell cycle and cell stemness (Fig. S2B,C). However, overexpression of RACGAP1P led to an enhanced migration ability (Fig. 2C–F) and invasion ability (Fig. 2G) in MDA‐MB‐231 RACGAP1P cells and MCF7 RACGAP1P cells. To further probe this oncogenic role of RACGAP1P *in vivo*, MDA‐MB‐231 RACGAP1P cells and MCF7 RACGAP1P cells were applied for tail vein injection xenograft tumor models. Eight weeks after tail vein injection in 4‐week‐old nude mice, RACGAP1P overexpression resulted in significantly increased lung metastasis foci (Fig. 2H,I). Collectively, these data demonstrate that RACGAP1P may not affect cell proliferation but play an essential role in breast cancer metastasis.

### 3.3 RACGAP1P‐induced mitochondrial fission is required for breast cancer cell invasion

**Fig. 3:**
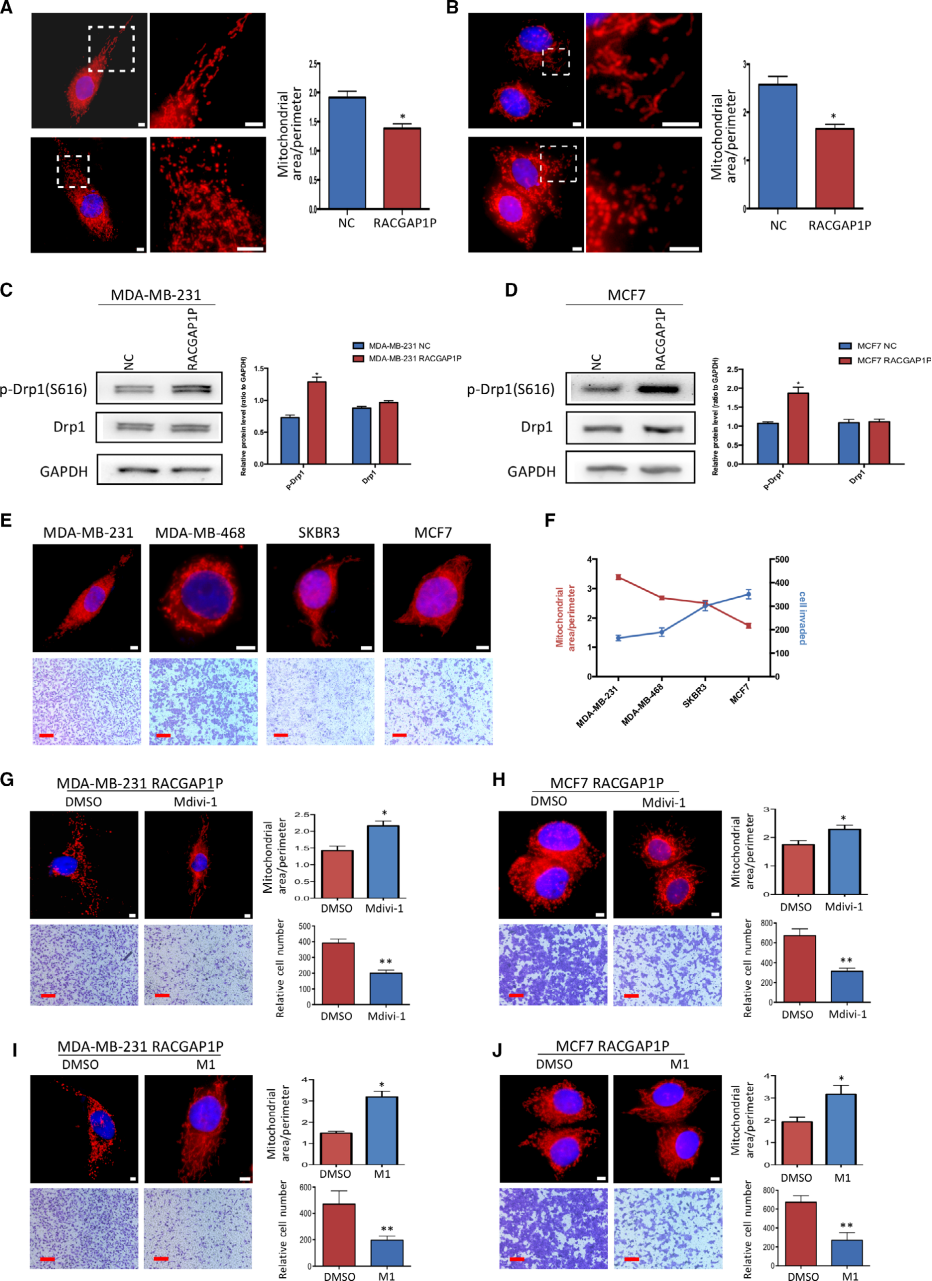
RACGAP1P promoted mitochondrial fission. (A and B) Mitochondrial morphology of MDA‐MB‐231 cells (A left) and MCF7 cells (B left) stably expressing scramble sequence and RACGAP1P stained by MitoTracker Red. Cells were analyzed using ImageJ to calculate mitochondrial area/perimeter ratios of individual cells (*n* = 20) as a measure of mitochondrial fission or fusion (A right and B right). Red, MitoTracker Red; Blue, DAPI. Scale bar, 5 μm. *P < 0.05. (C and D) Western blot analysis was used to detect the expression levels of Drp1 and p‐Drp1 in MDA‐MB‐231 and MCF7 cells. *P < 0.05. (E) Mitochondrial morphology staining and transwell assay (32 h after seeding) were performed with four different breast cancer cell lines. Scale bar (white), 5 μm; scale bar (red), 50 μm. (F) Mitochondrial dynamics and cell invasion abilities mentioned in (E) were quantized to analyze their relationship. (G and H) Mitochondrial morphology of RACGAP1P overexpressing MDA‐MB‐231 cells (G), MCF7 cells (H) treated with 0.1% DMSO or 25 μm Mdivi‐1 for 24 h and corresponding transwell assay (32 h for MDA‐MB‐231 RACGAP1P and 48 h for MCF7 RACGAP1P after seeding). Scale bar (white), 5 μm; Scale bar (red), 50 μm. *P < 0.05, **P < 0.01. (I and J) Mitochondrial morphology of RACGAP1P overexpressing MDA‐MB‐231 cells (I), MCF7 cells (J) treated with 0.1% DMSO or 5 μm M1 for 24 h and corresponding transwell assay (32 h for MDA‐MB‐231 RACGAP1P and 48 h for MCF7 RACGAP1P after seeding). Scale bar (white), 5 μm; scale bar (red), 50 μm. *P < 0.05, **P < 0.01. Error bars, SD; Student’s *t*‐test.

Previous studies showed that mitochondrial dynamics were implicated in cancer cell invasion [25]. We analyzed the mitochondrial morphology with MitoTracker Red staining in MDA‐MB‐231 and MCF7 cell lines. Overexpression of RACGAP1P in MDA‐MB‐231 cell line induced mitochondrial fission, which resulted in attenuated mitochondrial area/perimeter ratio (Fig. 3A). The same results were observed in MCF7 cell line (Fig. 3B). Moreover, we examined a mitochondrial fission protein Drp1 expression in MDA‐MB‐231 and MCF7 cell lines using Western blot. The results revealed that overexpression of RACGAP1P in both MDA‐MB‐231 cells and MCF7 cells led to promoted phosphorylation of Drp1‐S616, but did not increase the total Drp1 protein translation (Fig. 3C,D). To investigate the relationship between invasive ability and mitochondrial dynamics, we analyzed the cell invasive ability and mitochondrial morphology in four different breast cancer cell lines, including MDA‐MB‐231, MDA‐MB‐468, SKBR3, and MCF7. Consistent with previous studies [25], breast cancer cell lines with stronger invasive ability tended to have more mitochondrial fission than less invasive breast cancer cell lines (Fig. 3E,F). To further understand if mitochondrial network dynamics is responsible for breast cancer cell invasion, we treated MDA‐MB‐231 RACGAP1P cell line and MCF7 RACGAP1P cell line with Mdivi‐1, a mitochondrial fission inhibitor. We found mitochondria in both MDA‐MB‐231 RACGAP1P cells and MCF7 RACGAP1P cells treated with Mdivi‐1 shifted greatly from fragmentation to elongation following reduced invaded cell number compared to cells treated with DMSO (Fig. 3G,H). On the other hand, M1 is a mitochondrial fusion promotor [26]. After treating MDA‐MB‐231 RACGAP1P cells and MCF7 RACGAP1P cells with M1, we observed cell mitochondria shifted from fission to fusion as expected. More importantly, their invasive ability also dramatically reduced along with mitochondrial elongation (Fig. 3I,J). Together, these data suggest that RACGAP1P overexpression promotes breast cancer cell invasion via mitochondrial network reformation.

### 3.4 RACGAP1P regulates RACGAP1 expression by endogenously competitive binding with miR‐345‐5p

**Fig. 4:**
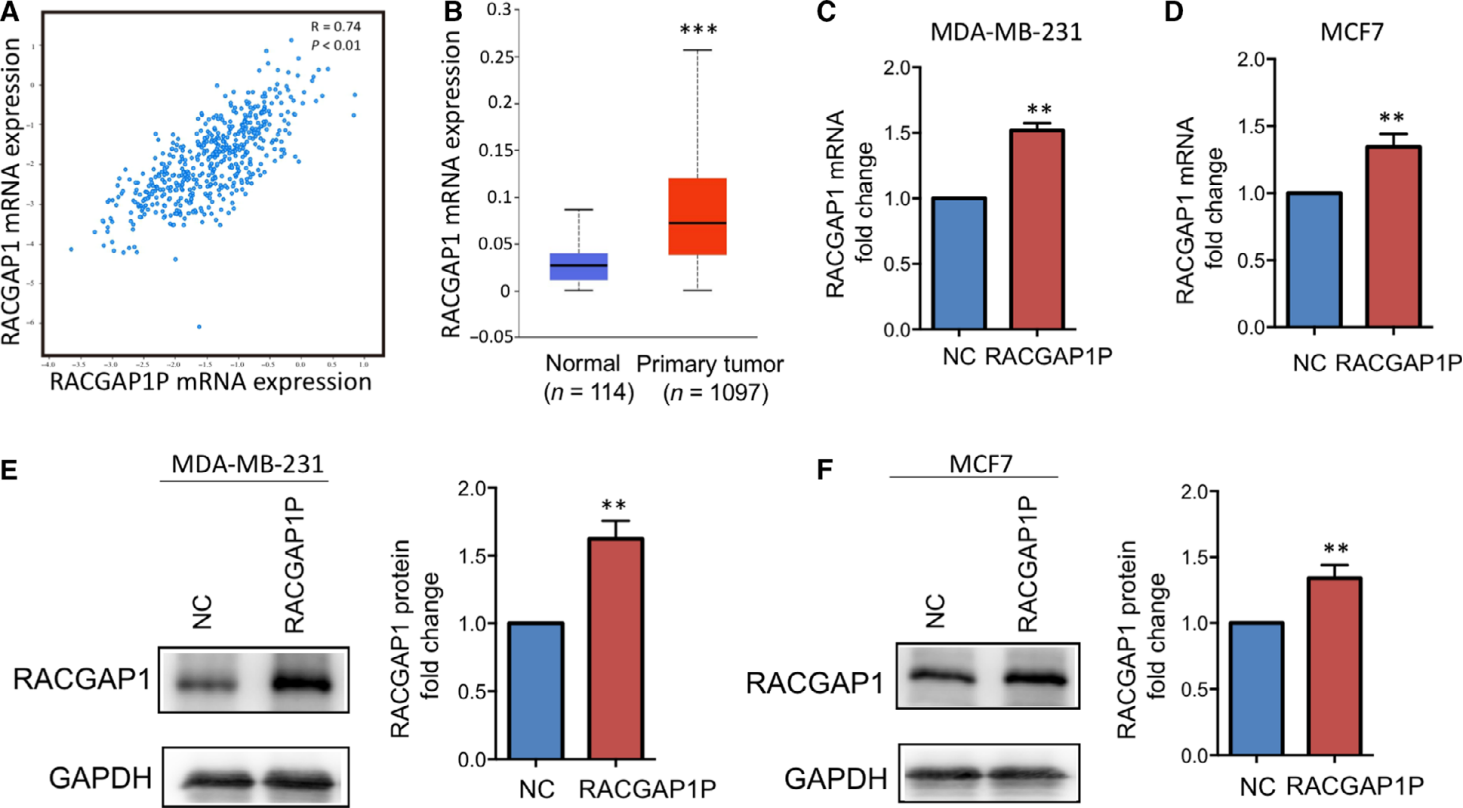
RACGAP1P was co‐expressed with RACGAP1 in breast cancer. (A) Co‐expression analysis of RACGAP1P and RACGAP1 in 460 microarray samples from TCGA dataset was visualized with cBioPortal. Pearson, *R* = 0.74. *P* < 0.01. (B)The mRNA expression level of RACGAP1 was revealed in breast cancer, performed on UALCAN online software. ***P < 0.001 (C and D) QRT‐PCR was performed to evaluate RACGAP1 mRNA expression in RACGAP1P overexpressing MDA‐MB‐231 and MCF7 cells. **P < 0.01. (E and F) Western blot was carried on to evaluate RACGAP1 protein expression in RACGAP1P overexpressed MDA‐MB‐231 and MCF7 cells. **P < 0.01.

**Fig. 5:**
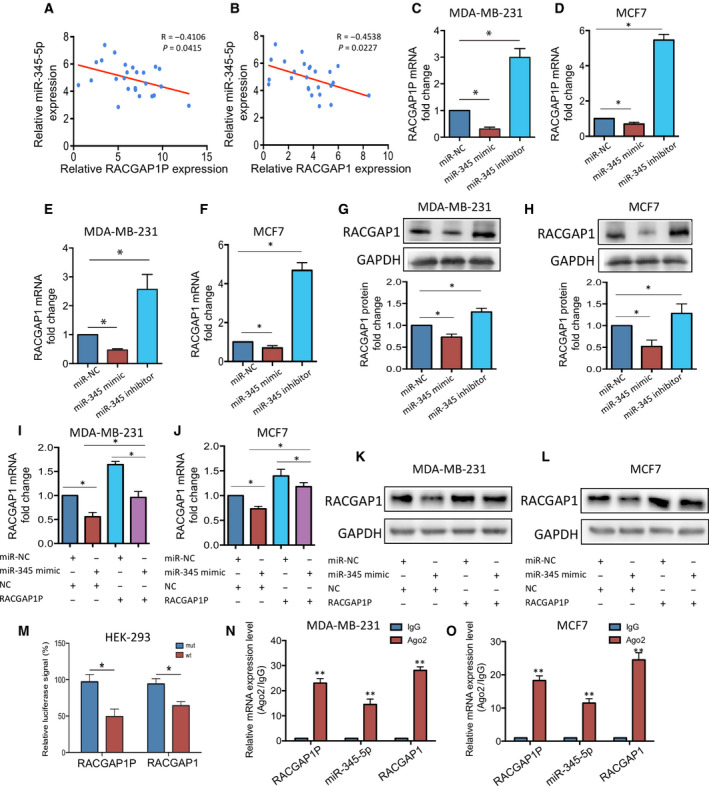
RACGAP1P acted as a ceRNA titrating miR‐345‐5p and up‐regulating RACGAP1. (A) The correlation between RACGAP1P and miR‐345‐5p was evaluated by Pearson analysis in 25 breast cancer tissues. *R* = −0.4106. *P* = 0.0415. (B) The correlation between RACGAP1 and miR‐345‐5p was evaluated by Pearson analysis in 25 breast cancer tissues. *R* = −0.4538. *P* = 0.0227. (C and D) QRT‐PCR was used to detect RACGAP1P expression in MDA‐MB‐231 and MCF7 cells transfected with scramble sequence, miR‐345‐5p mimic, or miR345‐5p inhibitor. *P < 0.05. (E‐H) QRT‐PCR (E‐F) and Western blot (G‐H) were carried on to evaluate RACGAP1 expression in MDA‐MB‐231 and MCF7 cells transfected with scramble sequence, miR‐345‐5p mimic or miR345‐5p inhibitor. *P < 0.05. (I‐L) The expression level of RACGAP was measured by qRT‐PCR (I and J) and Western blot (K and L) in MDA‐MB‐231 and MCF7 cells transfected with indicated DNA or RNA. (M) Dual‐Luciferase reporter assay was performed in MDA‐MB‐231 and MCF7 cells co‐transfected with miR‐345‐5p mimics and pMIR‐REPORT carrying wild‐type or mutant target sequence at 3′‐UTR of the reporter gene. *P < 0.05. (N and O) RIP assay was conducted in MDA‐MB‐231 and MCF7 cells to examine miR‐345‐5p endogenously associated with RACGAP1P and RACGAP1. **P < 0.01. Error bars, SD; Student’s *t*‐test.

Co‐expression analysis of 460 breast cancer microarray data from TCGA dataset showed that RACGAP1P expression level positively correlated with its parental gene RACGAP1 at the transcription level (*R* = 0.74, *P* < 0.01; Fig. 4A). RACGAP1 was confirmed to be overexpressed in breast cancer compared with normal tissues using data from TCGA (http://ualcan.path.uab.edu) (Fig. 4B). Overexpression of RACGAP1P resulted in up‐regulated RACGAP1 expression in MDA‐MB‐231 and MCF7 cells (Fig. 4C–F). The NCBI database was used to blast the sequence of RACGAP1P and RACGAP1, and we found that the transcripts of RACGAP1 and RACGAP1P are of high gene homology with identity of more than 92.72%. Therefore, we carried computational analysis with RegRNA2.0 [27], three miRNAs were identified, which were predicted to bind with RACGAP1P and RACGAP1 as well (Fig. S3A). Among them, miR‐345‐5p was reported to function as a tumor suppressor [28]. Pearson analysis shown that the expression level of miR‐345‐5p was negatively correlated with that of RACGAP1P or RACGAP1 (Fig. 5A,B). Thus, we hypothesized that RACGAP1P might function as a competitive endogenous RNA, sequestering RACGAP1 from miR345‐5p induced degradation. To test this hypothesis, the expression of RACGAP1P and RACGAP1 was analyzed with transfecting either miR345‐5p mimic or inhibitor. We found that overexpression of miR‐345‐5p significantly decreased both RACGAP1P and RACGAP1 expression in MDA‐MB‐231 and MCF7 cells, whereas knockdown of miR‐345‐5p significantly increased RACGAP1P and RACGAP1 expression (Fig. 5C–H). Moreover, co‐transfection of RACGAP1P and miR‐345‐5p showed that RACGAP1P reverted the down‐regulation of RACGAP1 in both mRNA and protein expression levels, mediated by miR‐345‐5p (Fig. 5I–L). Through microRNA target site scanning, miR‐345‐5p target sites were found at 1654–1673 nt of RACGAP1P and 1947–1966 nt of RACGAP1 (Fig. S3A). Dual‐luciferase reporter assay demonstrated that transfecting miR‐345‐5p greatly reduced luciferase activity of the reporter gene fused with wild‐type miR‐345‐5p target site at its the 3′UTR (Figs 5M and S3B). Besides, RIP assay confirmed the enrichment of RACGAP1P, miR‐345‐5p, and RACGAP1 in Ago2 precipitates (Fig. 5N,O). Collectively, these data indicate RACGAP1P regulates the expression of its parental gene RACGAP1 by endogenously competitive binding with miR‐345‐5p.

### 3.5 RACGAP1P promotes cell invasion ability dependent on RACGAP1

**Fig. 6:**
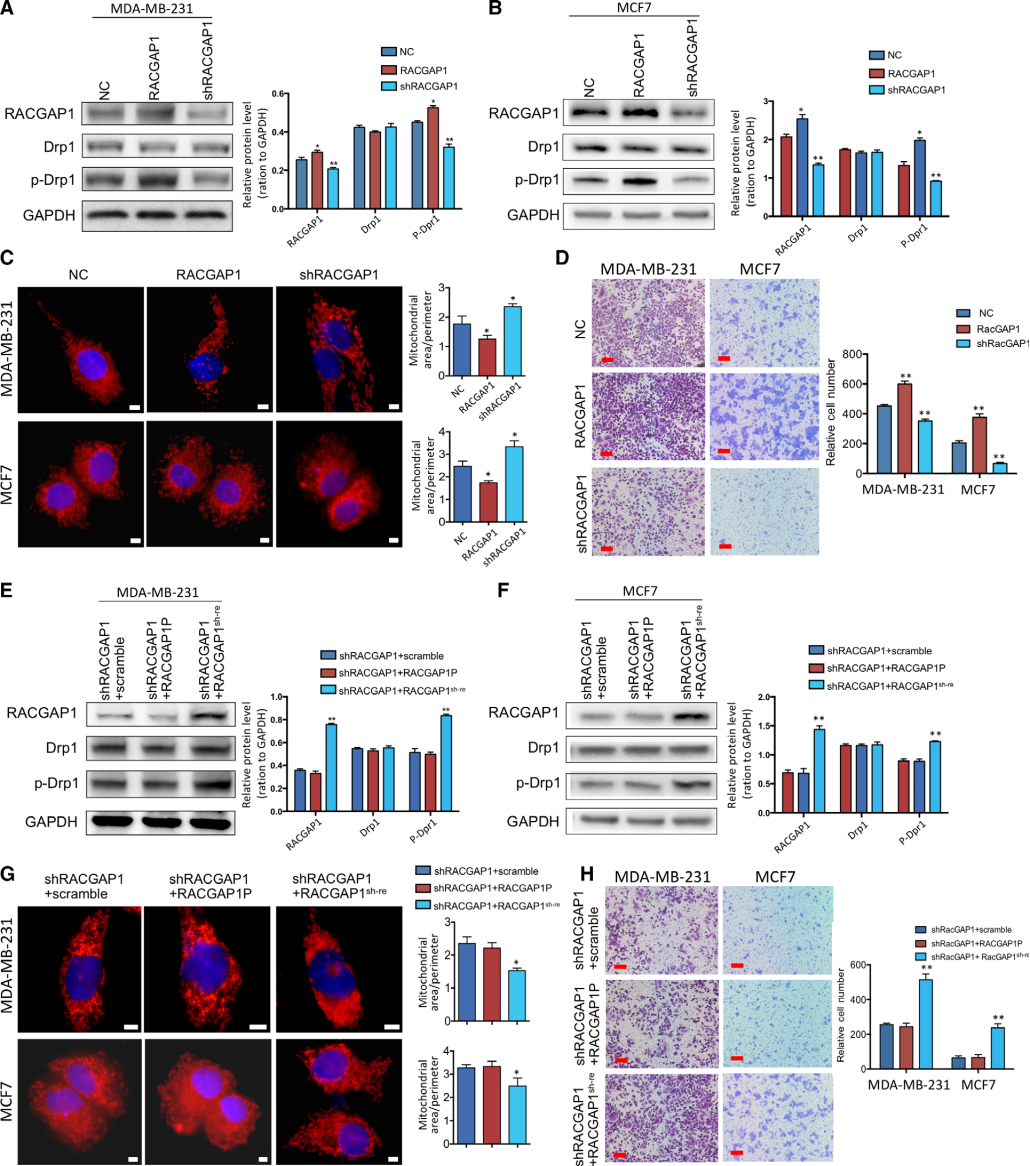
RACGAP1P‐induced cell invasion depended on RACGAP1. (A and B) Western blot analysis was used to detect the expression levels of RACGAP1, Drp1, and p‐Drp1 in MDA‐MB‐231 and MCF7 cells stably transfected with scramble sequence, RACGAP1 CDS, and RACGAP1 shRNA. *P < 0.05, **P < 0.01. (C and D) Mitochondrial morphology staining (C) and transwell assays (D) were performed in MDA‐MB‐231 and MCF7 cells stably transfected with scramble sequence, RACGAP1, and RACGAP1 shRNA. Scale bar (white), 5 μm; scale bar (red), 50 μm. *P < 0.05, **P < 0.01. (E and F) Western blot analysis was used to detect the expression levels of RACGAP1, Drp1, and p‐Drp1 in RACGAP1 knockdown MDA‐MB‐231 and MCF7 cells stably transfected with scramble sequence, RACGAP1P, and RACGAP1^sh‐re^. **P < 0.01. (G‐H) Mitochondrial morphology staining (G) and transwell assays (H) were performed in RACGAP1 knockdown MDA‐MB‐231 and MCF7 cells stably transfected with scramble sequence, RACGAP1P, and RACGAP1^sh‐re^. Scale bar (white), 5 μm; scale bar (red), 50 μm. *P < 0.05, **P < 0.01. Error bars, SD; Student’s *t*‐test.

As pseudogenes are highly possible to function through their parental genes [29], we hypothesized that RACGAP1 facilitated breast cancer cell invasion by inducing mitochondrial fragmentation. Stable RACGAP1 overexpression and knockdown MDA‐MB‐231 and MCF7 cell lines were established. Indeed, overexpression of RACGAP1 led to the activation of Drp1‐S616 and fragmented mitochondria, followed by enhanced invasive ability, whereas knockdown of RACGAP1 resulted in an opposite outcome (Fig. 6A‐D). To investigate whether RACGAP1P promotes cell invasion is RACGAP1‐dependent, we overexpressed RACGAP1P and shRNA‐resistant RACGAP1 constructs (RACGAP1^sh‐re^) in MDA‐MB‐231 and MCF7 cells expressing RACGAP1 shRNA. Interestingly, shRNA‐resistant RACGAP1 construct but not RACGAP1P rescued the mitochondria elongation induced invasive phenotype loss (Fig. 6E–H). We attempted to validate this result by knocking out the RACGAP1 gene using CRISPR/Cas9 system and overexpressing RACGAP1P or RACGAP1 in RACGAP1^−/−^ cells, but the absence of RACGAP1 led to cytokinesis dysfunction (Fig. S3C–E), RACGAP1^−/−^ cells were not able to survive after passage. Taken together, these data demonstrated that RACGAP1 was essential for RACGAP1P to exert the oncogenic properties.

## 4 Discussion

**Fig. 7:**
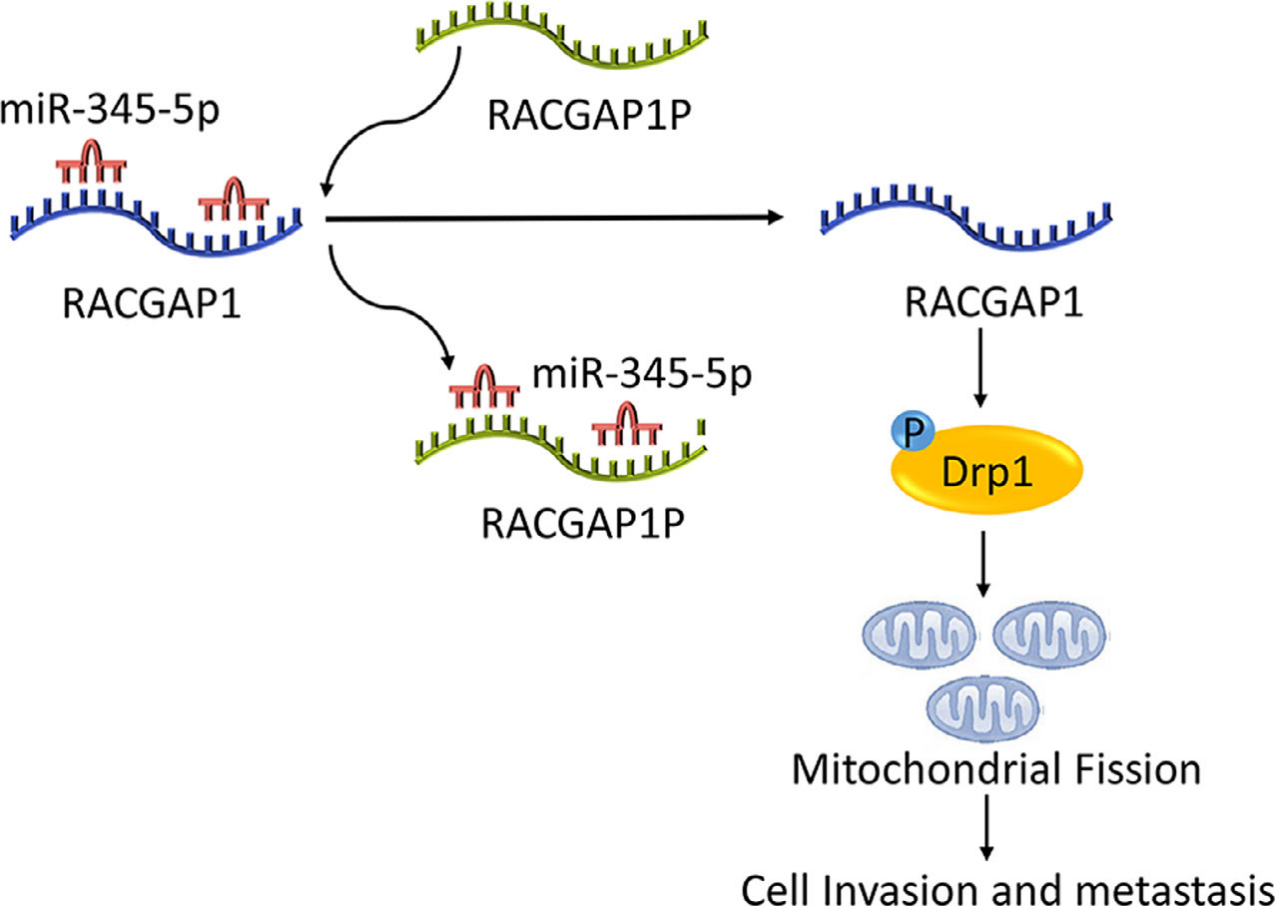
Potential mechanism of RACGAP1P implicated in breast cancer invasion and metastasis. In breast cancer cells, RACGAP1P could competitively bind to miR‐345‐5p that targeted RACGAP1 therefore up‐regulate RACGAP1. RACGAP1P overexpression promoted mitochondrial fission mediated cell invasion via RACGAP1P/miR‐345‐5p/RACGAP1/Drp1 network.

In this study, we identified one up‐regulated lncRNA, RACGAP1P, in the breast cancer tissue using LncRNA Expression Microarray, and its high expression was positively correlated with lymph node metastasis, distance metastasis, TNM stage, and shorter survival time. Further experiments showed that the overexpression of RACGAP1P could enhance mitochondrial fission by up‐regulating its parental gene RACGAP1 via competitively binding to miR‐345‐5p and thus increase the invasive ability of breast cancer cells. The results revealed the regulatory mechanism of RACGAP1P in the invasion and metastasis of breast cancer (Fig. 7).

LncRNAs have emerged to exhibit important biological function in cancer development and progression, involving in tumor cell proliferation [30], metabolism [31], migration, invasion [32], and even non‐coding RNA transfer [33, 34]. A previous study showed that overexpression of lncRNA RACGAP1P enhanced cell proliferation and migration, thus promoting hepatocellular carcinoma early recurrence [18]. Our results demonstrated that the up‐regulation of RACGAP1P was a commonly oncogenic event in breast cancer, which is correlated with lymph node metastasis, distance metastasis, TNM stage, and poor prognosis of breast cancer patients. Accordingly, functional experiments *in vivo* and *in vitro* confirmed that RACGAP1P promoted breast cancer cell migration, invasion, and metastasis, but had no significant effect on breast cancer cell proliferation. Consistently, we did not observe statistically significant relevance between RACGAP1P and tumor size in 102 breast cancer patients. As for the different effects on cell proliferation in hepatocellular carcinoma and breast cancer, we speculate that it may be due to the heterogeneity of breast cancer. Further investigation in cell biological behaviors involving RACGAP1P is needed.

Mitochondrial fission has been recognized as an important metastatic driver as the fragmentation of mitochondria helps the distribution of mitochondria by subcellular trafficking to the leading edge of migrating direction where energy is highly demanded to form lamellipodia during the process of migration and subsequent invasion [35]. Mitochondrial fragmentation is mainly regulated by the dynamin 1‐like protein (Drp1). Overexpression or enhanced activation of Drp1 mediates the mitochondrial fission in metastatic breast cancer [25], pancreatic cancer [9], and glioblastoma cells [36]. Drp1 activity is mainly regulated by post‐translational modifications, phosphorylation at serine (Ser) 616 residue site [37]. Moreover, several studies also showed that lncRNAs might play roles in mitochondrial function [38, 39, 40]. In this study, we found that the overexpression of RACGAP1P led to increased phosphorylation of Drp1‐S616 and promoted mitochondrial fission. While mitochondrial fission inhibitor Mdivi‐1 could diminish the invasive ability of RACGAP1P overexpression cells. Collectively, RACGAP1P promoted mitochondrial fission, which is required for breast cancer cell invasion via Drp1 activity enhancement.

Accumulating evidence suggests that RNA crosstalk plays a vital role in gene regulatory networks, and is implicated in cancer [41]. Pseudogenes are relicts of parental genes that lost the function of encoding for full‐length functional proteins during the evolutionary process [42]. Considering the homolog of pseudogenes with parental genes, pseudogenes have defined roles in regulating the expression of the parental genes through sequestering common miRNAs and releasing expression inhibition [43]. According to the NCBI database, RACGAP1P is the pseudogene of RACGAP1. Notably, RACGAP1P co‐expressed with RACGAP1 in breast cancer. Further experiments showed that RACGAP1P could regulate RACGAP1 expression by endogenously competitive binding with miR‐345‐5p. Previous studies demonstrated that RACGAP1 was a cancer‐promoting gene overexpressed in various cancers [44, 45, 46]. Importantly, RACGAP1 was identified as a metastatic driver in uterine carcinosarcoma [47]. Besides, RACGAP1 was also considered as a poor prognosis marker in high‐risk early breast cancer [48]. Consistent with these findings, we found that RACGAP1 could induce breast cancer cell invasion.

A great many of studies demonstrated RACGAP1 could enhance the phosphorylation level of Erk and activate Rho/Erk signaling [18, 49, 50]. Interestingly, the MAPK signal pathway was reported to be involved in mitochondrial fission that Erk1/2 could phosphorylate Drp1 on Ser616 [9]. Combined with our results, we speculate that RACGAP1P could promote mitochondrial fission through RACGAP1/Erk /Drp1 signaling axis.

## 5 Conclusion

In conclusion, overexpression of lncRNA RACGAP1P could enhance mitochondrial fission by competitive binding with miR‐345‐5p against its parental gene RACGAP1, thus enhancing the invasive and metastatic ability of breast cancer cells. RACGAP1P was an effective biomarker for the prognosis of breast cancer patients, and the blocking of RACGAP1P‐mediated mitochondrial fission might be a novel therapeutic target for metastatic breast cancer.

## Conflict of interest

The authors declare no conflict of interest.

## Author contributions

DMZ, KHR, and MLW performed all of the experiments and wrote the paper. JGW, EML, CJH, YS, YTJ, and QZ interpreted and discussed experimental data through the study. PZ and XPL supervised the entire study. All authors read and approved the final manuscript.

### Peer Review

The peer review history for this article is available at https://publons.com/publon/10.1002/1878‐0261.12866.

## Supporting information


**Fig. S1.** The structure of lentivirus vectors with all the sites specification.
**Fig. S2.** RACGAP1P was confirmed to be a lncRNA and had no significant effect on cell proliferation.
**Fig. S3.** The miRNA predicted to bind with RACGAP1P and RacGAP1.

**Table S1.** Clinicopathologic factors of five breast cancer patients included in microarray assay.

**Table S2.** The specifications of breast cancer cell lines.

**Table S3.** Primer sets used for qRT‐PCR.

**Table S4.** The sequence of shRNA‐resistant RACGAP1 cDNA.

**Table S5.** The shRNA sequence of RACGAP1.
