# Supplementary material for: Long non‐coding RNA RACGAP1P promotes breast cancer invasion and metastasis via miR‐345‐5p/RACGAP1‐mediated mitochondrial fission
Source: Mol Oncol. 2020 Dec 16;15(2):543–59. doi: 10.1002/1878-0261.12866 (PMC7858103; doi:10.1002/1878-0261.12866)
Supplement: Supplementary file 1 — Fig. S1. The structure of lentivirus vectors with all the sites specification. Fig. S2. RACGAP1P was confirmed to be a lncRNA and had no significant effect on cell proliferation. Fig. S3. The miRNA predicted to bind with RACGAP1P and RacGAP1. [file MOL2-15-543-s001.zip › mol212866-sup-0010-FigS2 legend.docx]

**Fig. S2.** RACGAP1P was confirmed to be a lncRNA and had no significant effect on cell proliferation.

(A) PhyloCSF analysis demonstrated the low possibility of protein coding ability within the 3 reading frame of RACGAP1P DNA sequence.

(B) Cell cycle distribution was analyzed with flowcytometry, 1 × 10^6^ cells were used per replication, assay was repeated 3 times. Error bars, mean with SD.

Student’s t-test.

(C) RACGAP1P overexpression did not significantly alter the CD44^+^/CD24^-^ cell population detected by flowcytometry. Error bars, SD; Two tailed Student’s t-test.
