# Supplementary figures and images for: Long non‐coding RNA RACGAP1P promotes breast cancer invasion and metastasis via miR‐345‐5p/RACGAP1‐mediated mitochondrial fission
Source: Mol Oncol. 2020 Dec 16;15(2):543–59. doi: 10.1002/1878-0261.12866 (PMC7858103; doi:10.1002/1878-0261.12866)

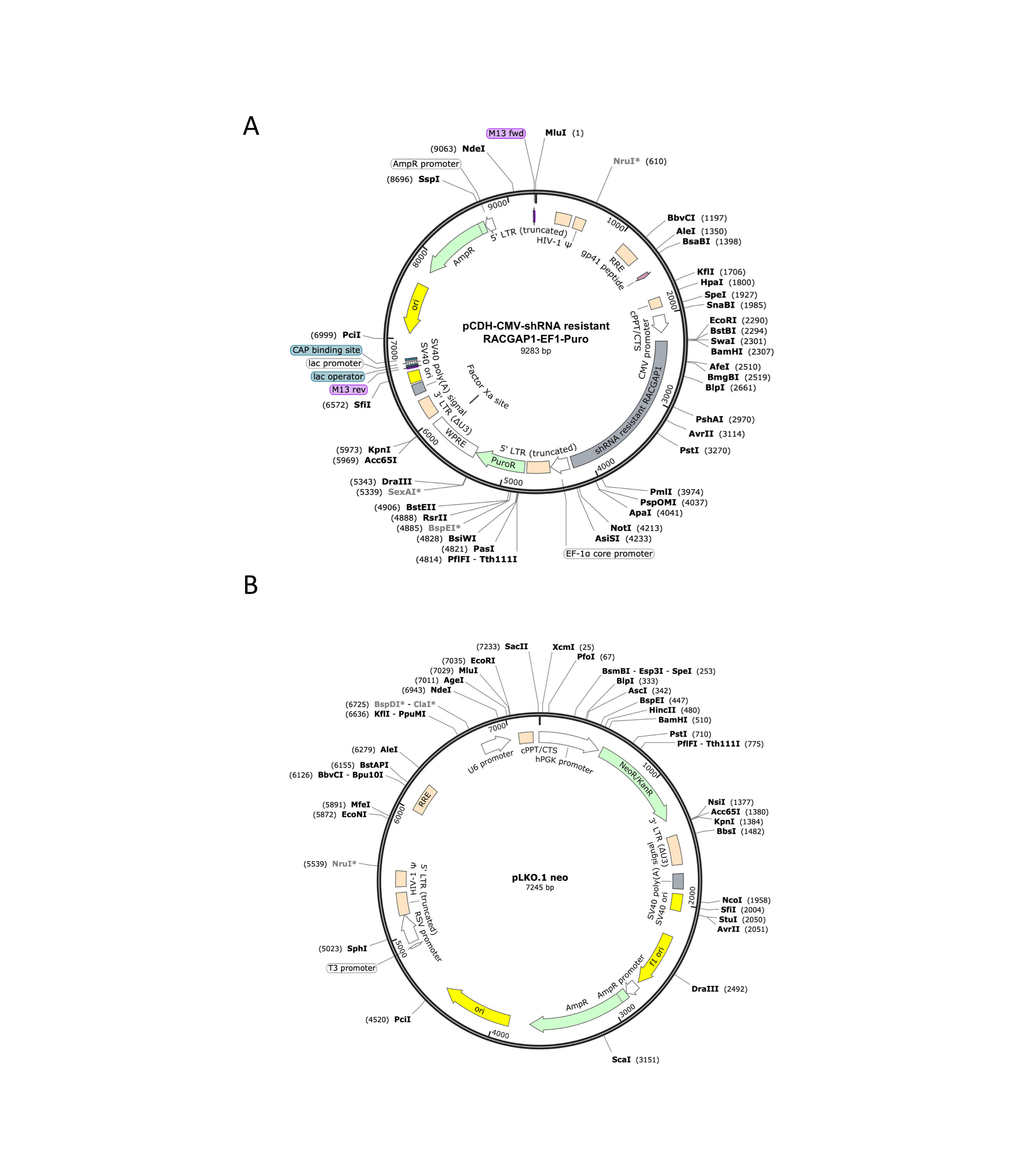

Supplement: Supplementary file 1 — Fig. S1. The structure of lentivirus vectors with all the sites specification. Fig. S2. RACGAP1P was confirmed to be a lncRNA and had no significant effect on cell proliferation. Fig. S3. The miRNA predicted to bind with RACGAP1P and RacGAP1. [file MOL2-15-543-s001.zip › mol212866-sup-0001-FigS1.tif]

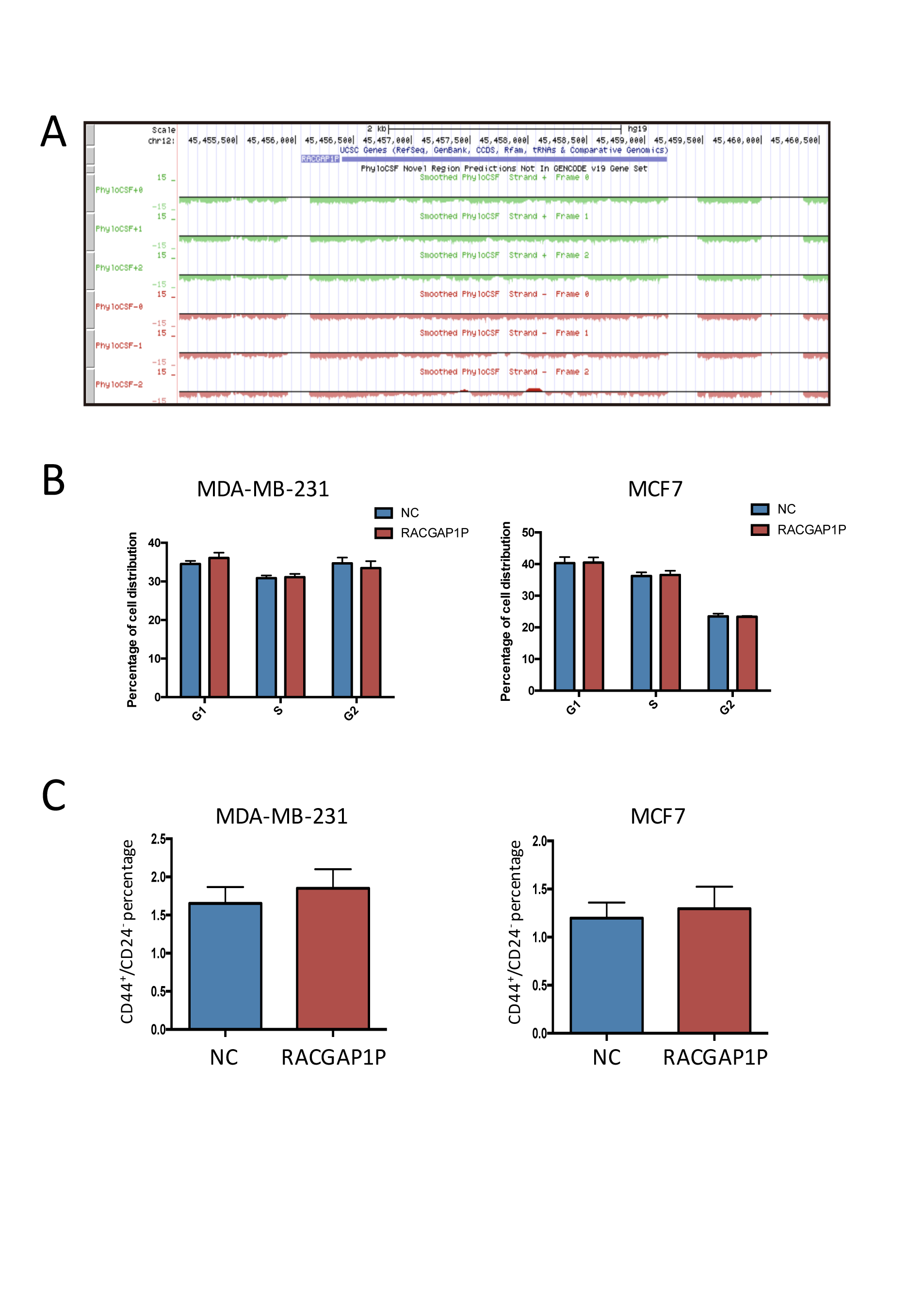

Supplement: Supplementary file 1 — Fig. S1. The structure of lentivirus vectors with all the sites specification. Fig. S2. RACGAP1P was confirmed to be a lncRNA and had no significant effect on cell proliferation. Fig. S3. The miRNA predicted to bind with RACGAP1P and RacGAP1. [file MOL2-15-543-s001.zip › mol212866-sup-0002-FigS2.tif]

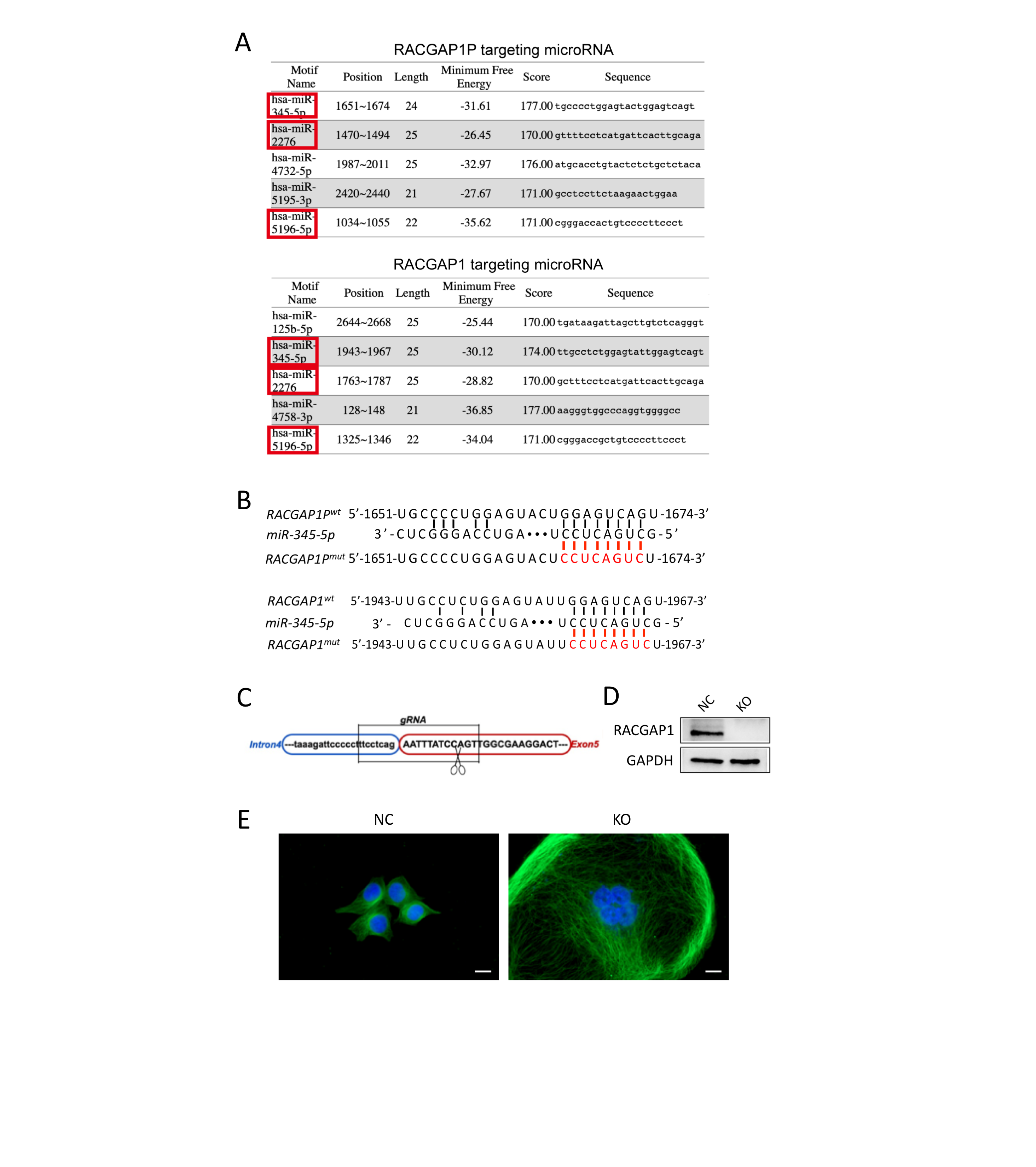

Supplement: Supplementary file 1 — Fig. S1. The structure of lentivirus vectors with all the sites specification. Fig. S2. RACGAP1P was confirmed to be a lncRNA and had no significant effect on cell proliferation. Fig. S3. The miRNA predicted to bind with RACGAP1P and RacGAP1. [file MOL2-15-543-s001.zip › mol212866-sup-0003-FigS3.tif]
