# Supplementary material for: Long non‐coding RNA RACGAP1P promotes breast cancer invasion and metastasis via miR‐345‐5p/RACGAP1‐mediated mitochondrial fission
Source: Mol Oncol. 2020 Dec 16;15(2):543–59. doi: 10.1002/1878-0261.12866 (PMC7858103; doi:10.1002/1878-0261.12866)
Supplement: Supplementary file 2 — Table S1. Clinicopathologic factors of five breast cancer patients included in microarray assay. [file MOL2-15-543-s002.docx]

**Table S1.** Clinicopathologic factors of five breast cancer patients included in microarray assay.

| Characteristics | No.1 | No.2 | No.3 | No.4 | No.5 |
| --- | --- | --- | --- | --- | --- |
| Gender | Female | Female | Female | Female | Female |
| Age (years) | 41 | 45 | 51 | 54 | 61 |
| Tumor size (cm) | 4 | 3.5 | 4.5 | 3 | 3.5 |
| Pathological diagnosis | Invasive breast cancer, no specific type | Invasive breast cancer, no specific type | Invasive breast cancer, no specific type | Invasive breast cancer, no specific type | Invasive breast cancer, no specific type |
| ER status | - | - | - | + | + |
| PR status | - | - | - | + | + |
| HER2 status | 3+ | 3+ | 1+ | 1+ | 1+ |
| Ki67 | 40%+ | 50%+ | 70%+ | 15%+ | 50%+ |
| Subtype | HER2-positive | HER2-positive | Triple negative | Luminal A | Luminal B |
