# Supplementary material for: Long non‐coding RNA RACGAP1P promotes breast cancer invasion and metastasis via miR‐345‐5p/RACGAP1‐mediated mitochondrial fission
Source: Mol Oncol. 2020 Dec 16;15(2):543–59. doi: 10.1002/1878-0261.12866 (PMC7858103; doi:10.1002/1878-0261.12866)
Supplement: Supplementary file 3 — Table S2. The specifications of breast cancer cell lines. [file MOL2-15-543-s003.docx]

| cell | ER status | PR status | HER2 status | aggressiveness |
| --- | --- | --- | --- | --- |
| T47D | + | + | - | low |
| MCF7 | + | + | - | low |
| MDA-MB-468 | - | - | - | high |
| MDA-MB-453 | - | - | + | low |
| BT474 | + | + | + | low |
| BT549 | - | - | - | high |
| MDA-MB-231 | - | - | - | high |

**Table S2.** The specifications of breast cancer cell lines
