# Supplementary material for: Long non‐coding RNA RACGAP1P promotes breast cancer invasion and metastasis via miR‐345‐5p/RACGAP1‐mediated mitochondrial fission
Source: Mol Oncol. 2020 Dec 16;15(2):543–59. doi: 10.1002/1878-0261.12866 (PMC7858103; doi:10.1002/1878-0261.12866)
Supplement: Supplementary file 4 — Table S3. Primer sets used for qRT‐PCR. [file MOL2-15-543-s004.docx]

**Table S3**. Primer sets used for qRT-PCR.

|  |  | Sequence (5′-3′) |
| --- | --- | --- |
| RACGAP1P | Forward | AGGTGCAGAGCAAAGTGAAG |
|  | Reverse | TTTTCAGTCAGCCTGGCCTC |
| RACGAP1 | Forward | GAGCCCAACTTCTAAAGGTCA |
|  | Reverse | ATTTCAGCATCCAAAGTGCAA |
| GAPDH | Forward | CTGACTTCAACAGCGACACC |
|  | Reverse | TGCTGTAGCCAAATTCGTTGT |
| RACGAP1P | 1-Forward | ATATCTCCCCTTCCTTCTCC |
|  | 1-Reverse | CAACAGTGACCAGAACAAGG |
| RACGAP1P | 2-Forward | TCACTGGATTGCGACTCTTC |
|  | 2-Reverse | TTTCTCTCTGCTTCCGCTTG |
| RACGAP1P | 3-Forward | CAGTTTGTTGATGGTCCCTC |
|  | 3-Reverse | TGTGGAGCCAATGGAACGAG |
| GAPDH | 1-Forward | AAAAGGGCCCTGACAACTCT |
|  | 1-Reverse | TGCTGTAGCCAAATTCGTTG |
| GAPDH | 2-Forward | CCTTCATTGACCTCAACTAC |
|  | 2-Reverse | GGGTGGAATCATATTGGAAC |
| GAPDH | 3-Forward | GCCTCACTCCTTTTGCAGAC |
|  | 3-Reverse | ACAGTCTTCTGGGTGGCAGT |
| RACGAP1P, RACGAP1, and GAPDH Primer were designed to amplify RNA in cell lines and 25 paired of breast cancer tissues.  RACGAP1P-1，2，3 and GAPDH-1，2，3 Primer were designed to amplify RNA in 102 FFPE breast cancer tissues. | | |
