# Supplementary material for: Long non‐coding RNA RACGAP1P promotes breast cancer invasion and metastasis via miR‐345‐5p/RACGAP1‐mediated mitochondrial fission
Source: Mol Oncol. 2020 Dec 16;15(2):543–59. doi: 10.1002/1878-0261.12866 (PMC7858103; doi:10.1002/1878-0261.12866)
Supplement: Supplementary file 5 — Table S4. The sequence of shRNA‐resistant RACGAP1 cDNA. [file MOL2-15-543-s005.docx]

| ATGGATACCATGATGCTCAACGTGAGAAATCTGTTCGAGCAGCTCGTGAGAAGAGTCGAGATTCTGAGCGAAGGCAACGAGGTCCAATTCATCCAGCTGGCCAAGGATTTCGAGGATTTCAGAAAGAAGTGGCAAAGAACCGACCACGAGCTGGGCAAGTACAAGGATCTGCTGATGAAAGCCGAGACAGAGAGGAGCGCTCTCGACGTGAAGCTGAAGCATGCTAGAAACCAAGTGGATGTGGAGATCAAGAGGAGACAAAGAGCCGAAGCCGATTGTGAGAAGCTGGAGAGGCAGATCCAGCTGATTAGAGAGATGCTCATGTGTGACACAAGCGGCAGCATCCAGCTGAGCGAGGAACAGAAGTCCGCCCTCGCCTTTCTGAATAGAGGCCAGCCTAGCAGCTCCAACGCCGGCAACAAAAGACTCAGCACCATCGATGAGTCCGGCTCCATTCTGAGCGACATCTCCTTCGACAAGACCGACGAATCTCTGGACTGGGACAGCTCTCTGGTGAAGACCTTTAAGCTGAAGAAGAGAGAGAAGAGGAGAAGCACCTCTAGACAGTTTGTCGACGGACCCCCCGGCCCCGTCAAGAAAACAAGAAGCATCGGCAGCGCCGTCGACCAAGGCAACGAGTCCATTGTGGCCAAGACCACAGTCACAGTGCCCAACGATGGCGGCCCCATCGAGGCTGTCTCCACCATTGAGACAGTGCCCTACTGGACAAGATCTAGAAGAAAGACCGGAACACTGCAACCTTGGAACTCCGACAGCACCCTCAACTCCAGACAGCTGGAGCCTAGGACCGAGACAGACTCCGTGGGAACCCCCCAAAGCAATGGCGGAATGAGACTGCACGACTTCGTGTCCAAGACCGTCATTAAGCCCGAAAGCTGCGTGCCTTGCGGCAAGAGGATCAAATTCGGCAAGCTCTCTCTGAAGTGTAGAGACTGCAGAGTGGTGTCCCACCCCGAGTGTAGAGATAGATGCCCCCTCCCTTGCATCCCCACACTCATCGGCACCCCCGTCAAAATCGGCGAGGGCATGCTCGCTGATTTTGTGAGCCAGACCAGCCCCATGATCCCCTCCATTGTGGTGCACTGCGTCAACGAGATCGAGCAGAGAGGACTGACAGAGACCGGACTGTATAGAATCAGCGGCTGCGACAGAACCGTGAAGGAGCTCAAGGAGAAGTTTCTGAGGGTGAAGACAGTGCCTCTGCTGTCCAAGGTGGACGATATTCACGCCATCTGTTCTCTGCTGAAGGACTTTCTGAGGAATCTGAAGGAGCCTCTGCTGACCTTCAGACTGAATAGAGCCTTTATGGAGGCCGCCGAGATTACCGACGAGGACAACTCCATCGCCGCTATGTATCAAGCCGTGGGCGAGCTGCCTCAAGCCAATAGAGACACACTCGCCTTTCTGATGATCCACCTCCAAAGAGTGGCCCAATCCCCTCATACCAAGATGGACGTCGCTAATCTGGCCAAAGTGTTCGGCCCTACCATTGTGGCCCACGCTGTCCCCAATCCCGATCCCGTGACCATGCTCCAAGACATCAAGAGGCAGCCTAAGGTGGTGGAGAGACTCCTCTCCCTCCCTCTGGAGTACTGGTCCCAGTTCATGATGGTGGAACAAGAGAACATCGACCCTCTGCACGTGATCGAGAATAGCAACGCCTTCAGCACCCCTCAAACCCCCGACATTAAGGTGTCTCTGCTGGGCCCCGTCACAACACCCGAGCATCAGCTGCTGAAGACCCCCTCCAGCAGCTCTCTGAGCCAAAGAGTGAGAAGCACCCTCACCAAAAACACCCCTAGATTCGGAAGCAAGAGCAAGTCCGCCACCAATCTGGGAAGGCAAGGCAACTTTTTCGCTAGCCCCATGCTGAAATGA |
| --- |

**Table S4.** The sequence of shRNA-resistant RACGAP1 cDNA (5′-3′)
