# Supplementary material for: Long non‐coding RNA RACGAP1P promotes breast cancer invasion and metastasis via miR‐345‐5p/RACGAP1‐mediated mitochondrial fission
Source: Mol Oncol. 2020 Dec 16;15(2):543–59. doi: 10.1002/1878-0261.12866 (PMC7858103; doi:10.1002/1878-0261.12866)
Supplement: Supplementary file 6 — Table S5. The shRNA sequence of RACGAP1. [file MOL2-15-543-s006.docx]

**Table S5.** The shRNA sequence of RACGAP1

| Sense (5′-3′) | containing enzyme cutting site of Agel and EcoRI | CCGGACGGCGCGGGAGGAATAAATTCTCGAGAATTTATTCCTCCCGCGCCGTTTTTTG |
| --- | --- | --- |
| Antiense (5′-3′) | containing enzyme cutting site of Agel and EcoRI | AATTCAAAAAACGGCGCGGGAGGAATAAATTCTCGAGAATTTATTCCTCCCGCGCCGT |
